# The regulatory role of immune microenvironment-related cells and pathways in the pathogenesis of keloids

**DOI:** 10.3389/fimmu.2025.1529564

**Published:** 2025-07-11

**Authors:** Xuan Dong, Mingnan Gao, Han Guo, Peng Wang, Yixuan Zhang, Qiaoli Shang, Qiying Wang

**Affiliations:** Department of Plastic Surgery, First Affiliated Hospital of Zhengzhou University, Zhengzhou, Henan, China

**Keywords:** keloids, immune microenvironment, cytokines, signaling pathways, regulatory

## Abstract

Keloids are skin lesions caused by excessive fibrotic reactions, and their pathogenesis is not yet fully understood. Recent studies have shown that the immune microenvironment plays a significant role in the development of keloids. This article reviews the distribution and functions of immune microenvironment-related cells in keloids, including keratinocytes, fibroblasts, mast cells, macrophages, T cells, and stem cells, as well as the interactions between these cells and local cells. The article also explores the impact of several signaling pathways within the immune microenvironment on keloid formation, including the transforming growth factor β pathway (TGF-β), PI3K/Akt/mTOR signaling pathway, Wnt/β-catenin signaling pathway, and Notch signaling pathway. These pathways recruit more immune cells by secreting various cytokines and inflammatory mediators, stimulate fibroblast proliferation and collagen synthesis, ultimately leading to the formation of keloids. By deeply analyzing the roles of cells and their signaling pathways within the immune microenvironment, we can provide potential new targets for the treatment of keloids.

## 1 Introduction

Keloids are benign tumor-like lesions formed by the abnormal proliferation of fibrous tissue on the skin. They are characterized by persistent scar formation that often extends beyond the original site of injury and is accompanied by excessive collagen deposition. The occurrence of this condition is typically due to an abnormal increase in fibroblast proliferation and collagen synthesis during the healing process following skin injury, leading to excessive scarring. Keloids not only affect the patient’s appearance but may also be associated with discomfort such as pain and itching, and in severe cases, they can impact function and quality of life, increasing psychological stress for patients (1). Genetic factors along with individual and environmental factors play a significant role in the formation of keloids. Studies indicate that the prevalence of keloids is highest in people of African ethnicity, followed by Asians and Hispanics, with the lowest prevalence in individuals of white ethnicity (2). Keloids can occur at any age but are most common in young people (3). The prevalence of keloids varies across different anatomical sites, and the condition is more common in adolescents and pregnant women, who exhibit increased hormonal activity and pronounced skin pigmentation (4). These observations suggest a close association between keloid formation and melanin pigmentation.

In China, keloids are prevalent, often appearing on areas such as the earlobes, chest, and shoulders. However, the exact pathogenesis remains unclear, making treatment challenging (5). Currently, various treatment methods are available, including surgery, radiation therapy, corticosteroid injections, silicone gel sheets, and pressure therapy. However, their effectiveness is often suboptimal, requiring long treatment courses, being expensive, and presenting notable side effects (6). Therefore, exploring the mechanisms underlying keloid formation and identifying more reliable targets for prevention and treatment is particularly important.

In recent years, the role of immune microenvironment-related cells and pathways in the mechanisms of keloid formation has received increasing attention, and they are considered to play a key role in the pathogenesis of keloids. Cells within the immune microenvironment, including keratinocytes, fibroblasts, mast cells, macrophages, T cells, and stem cells, play critical roles. These cells participate in various aspects of immune regulation during the development of keloids, such as the release of inflammatory mediators (e.g., interferon-γ(IFN-γ), IL-2, IL-6, TNF-α, IL-6, IL-10), as well as cell proliferation and migration (7, 8). The activation state of immune cells and the level of inflammation during the wound healing process directly influence scar formation and tissue repair. In addition to cells, several related signaling pathways have also been confirmed to play crucial roles in the pathogenesis of keloids, including TGF-βsignaling pathway, PI3K/Akt/mTOR signaling pathway, Wnt/β-catenin signaling pathway, and Notch signaling pathway. These signaling pathways affect the formation and repair of scars by regulating inflammatory responses, cell proliferation, matrix synthesis, and innate immunity. In-depth research on these signaling pathways can provide important insights into the molecular mechanisms underlying scars and potential therapeutic strategies.

We review the latest research findings on the role of immune microenvironment-related cells and pathways in the pathogenesis of keloids, aiming to gain a more comprehensive understanding of the regulatory mechanisms involved in the formation of keloids. Additionally, this will pave the way for the application of immune-related therapeutic approaches in the treatment of keloids.

## 2 The mechanisms of keloid formation

The pathogenesis of keloids is highly complex, the specific mechanisms remain unclear and it is the result of a combination of various factors. Currently, it is believed that the formation of keloids is primarily associated with abnormal proliferation of fibroblasts, excessive collagen synthesis, infiltration of inflammatory cells, and accumulation of extracellular matrix (4, 9, 10).During the wound healing process, normal fibrotic responses typically cease. However, in keloids, fibroblasts continue to proliferate and produce excess type I and type III collagen, leading to the progressive enlargement of scar tissue. Keloid fibroblasts are generally regarded as the primary cell type involved in keloid formation, but the regulatory mechanisms of their immune microenvironment have not been fully studied. This article will review the role of immune microenvironment-related cells and pathways in the pathogenesis of keloids, with the aim of providing new insights for the treatment of keloids.

## 3 Immune microenvironment-related cells in keloids

### 3.1 Keratinocytes

Keratinocytes are the main constituent cells of the epidermis. During skin injury and healing, keratinocytes not only participate in barrier repair but also regulate immune responses and the activation of fibroblasts by secreting various cytokines and growth factors (11). They can recognize pathogen-associated molecular patterns (PAMPs) and damage-associated molecular patterns (DAMPs), thereby initiating a series of immune responses.

#### 3.1.1 The immune regulatory role of keratinocytes in keloids

In keloids, keratinocytes exhibit abnormal patterns of cytokine and chemokine secretion. These factors include interleukins (ILs), tumor necrosis factor (TNF), and transforming growth factor (TGF), among others. The aberrant secretion of these factors not only promotes the proliferation and differentiation of keratinocytes themselves but also attracts and activates immune cells such as macrophages and neutrophils, further exacerbating the inflammatory response and fibrotic process in keloids. As non-professional antigen-presenting cells, keratinocytes also demonstrate significant changes in their antigen presentation function in keloids. By expressing major histocompatibility complex (MHC) molecules and co-stimulatory molecules, they can effectively present antigens to T cells, thereby initiating adaptive immune responses (12, 13). In keloids, keratinocytes can activate T cells by presenting self-antigens or pathogen-derived antigens, leading to the secretion of various cytokines such as IFN-γ and IL-17. These cytokines further promote the formation and development of keloids. This abnormal antigen presentation function may result in an imbalance of the local immune microenvironment in keloids, facilitating their formation and progression.

#### 3.1.2 The interaction between keratinocytes and the immune microenvironment in keloids

In the immune microenvironment of keloids, there is a close interaction between keratinocytes and immune cells. On one hand, keratinocytes attract and activate immune cells by secreting chemokines; on the other hand, inflammatory mediators released by immune cells in turn influence the biological behavior of keratinocytes. This interaction forms a complex immune network that collectively regulates the occurrence and development of keloids.

The extracellular matrix (ECM) is a major component of fibrotic tissue in keloids. Keratinocytes participate in the degradation and remodeling of the ECM by secreting various matrix metalloproteinases (MMPs) and tissue inhibitors of metalloproteinases (TIMPs) (14). In keloids, the imbalance between MMPs and TIMPs secreted by keratinocytes leads to excessive ECM deposition and exacerbated fibrosis.

Keratinocytes play a key role in regulating local immune responses by forming physical barriers and secreting various cytokines, including pro-inflammatory and anti-inflammatory factors. They can express co-stimulatory molecules and participate in antigen presentation, thereby activating T cells. Additionally, keratinocytes induce the migration of immune cells to the site of injury by releasing chemotactic factors, facilitating wound healing. In some cases, they can also achieve local immune tolerance through the expression of immune checkpoint molecules.

### 3.2 Fibroblasts

Fibroblasts play a key role in the formation of keloids by promoting the formation of scar tissue through the synthesis of collagen and extracellular matrix, signal transduction, inflammation regulation, and interaction with other cells (15).

Fibroblasts primarily originate from the dermis and are a highly plastic cell type responsible for synthesizing and remodeling the ECM as well as regulating tissue repair. During normal healing processes, fibroblasts promote wound healing by migrating to the injury site, proliferating, and secreting components such as collagen. However, in keloids, this normal process is disrupted, leading to uncontrolled activation and proliferation of fibroblasts, resulting in excessive deposition of collagen (16).

Fibroblasts play a significant immune role in the formation and development of keloids. In the pathological process of keloids, fibroblasts are not only responsible for the synthesis of collagen and other matrix components but also participate in the local inflammatory response. They promote the infiltration and activation of immune cells by secreting various cytokines and chemokines (such as IL-6, TGF-β, PDGF, etc.), thereby exacerbating local inflammation. These factors promote the infiltration and activation of immune cells, thereby exacerbating local inflammation. The release of these factors further stimulates the proliferation of fibroblasts and their collagen-synthesizing ability, creating a positive feedback loop that leads to continual proliferation of scar tissue. Additionally, fibroblasts can modulate the function of immune cells, affecting their ability to regenerate and repair scar tissue. Therefore, during the development of keloids, fibroblasts are not only structural components but also important factors in regulating immune responses and fibrosis.

### 3.3 Immune cells

#### 3.3.1 Macrophages

Macrophages are a crucial immune component that plays a dual role in tissue repair and fibrosis. Scar is a pathological scar characterized by excessive deposition of collagen and activation of fibroblasts. Macrophages regulate the behavior of fibroblasts and reshape the local microenvironment (17). During early wound healing, they rapidly infiltrate injury sites, clearing debris/pathogens while secreting cytokines to initiate repair processes. These factors include pro-inflammatory cytokines (such as IL-1, IL-6, TNF-α) and growth factors (such as TGF-β, vascular endothelial growth factor (VEGF), etc.). The release of these factors not only attracts more immune cells but also stimulates the proliferation of fibroblasts and collagen synthesis (18). Macrophages also modulate extracellular matrix composition via MMPs, influencing keloid formation (19). Keloid tissue shows increased macrophage infiltration, predominantly M2-polarized cells. These pro-repair, anti-inflammatory M2 macrophages promote fibrosis by secreting growth factors (e.g., TGF-β) that stimulate fibroblast activity and proliferation (20).These findings demonstrate macrophages’ dual role in keloid pathogenesis: participating in initial inflammation while directly modulating fibroblast function through polarization. Importantly, a bidirectional crosstalk exists between macrophages and fibroblasts. Keloid fibroblasts not only respond to macrophage signals but also secrete TGF-β to promote M2 polarization (21), creating a pro-fibrotic feedback loop that exacerbates scarring (22).This macrophage-fibroblast crosstalk critically drives keloid progression through both direct cellular interactions and modulation of the local immune microenvironment (23).Research indicates that macrophages can attract other immune cells (such as lymphocytes and neutrophils) to the site of injury by releasing pro-inflammatory factors. The involvement of these cells can further enhance the local inflammatory response, and sustained inflammation may promote keloid formation (24). Therefore, altering the polarization state of macrophages or inhibiting their pro-inflammatory responses could represent a new strategy for treating keloids.

#### 3.3.2 T cell

T cells are a type of lymphocyte produced in the thymus, playing a crucial role in the immune system. Based on their surface markers and functions, T cells can be primarily divided into two major categories: CD4^+^ T cells and CD8^+^ T cells. CD4^+^ T cells can further be subdivided into different subsets, such as Th1 (helper T cells type 1), Th2 (helper T cells type 2), and Th17 (helper T cells type 17). Each subset has unique functions in regulating immune responses and mediating inflammation.

T cells play an important role in modulating immune responses, participating in inflammatory reactions, and contributing to fibrotic mechanisms. Research indicates that T cells influence the occurrence and development of keloids through various mechanisms.

After skin injury, local tissue damage triggers a series of inflammatory responses. The cytokines and chemokines released from the damaged area can induce T cells to migrate from the bloodstream to the site of injury. In this process, the initial immune response primarily relies on the actions of macrophages and dendritic cells (DCs), which effectively capture pathogens and present antigens, thereby activating T cells (25).

Activated T cells begin to proliferate and migrate toward the damaged area, a process regulated by various cytokines, including IL-2, IL-6, and TGF-β. In keloids, the accumulation and activity of CD4^+^ T cells are particularly considered to be closely related to changes in the local microenvironment (26).

Research shows that the number of CD4^+^ T cells significantly increases in keloid tissue, and these cells promote the proliferation of fibroblasts and the synthesis of collagen by secreting cytokines such as IL-6 and IL-13 (27). This finding suggests the importance of Th2-type immune responses in keloid formation, and the release of Th2 cell cytokines may induce fibroblasts to transform into myofibroblasts, thereby exacerbating the fibrotic process.

Additionally, CD8^+^ T cells also play a complex role in the formation of keloids. Although CD8^+^ T cells are generally associated with host defense against infections and tumor surveillance, research has shown that their functions in keloid formation should not be overlooked. Some studies suggest that CD8^+^ T cells can inhibit the proliferation of fibroblasts and collagen synthesis by secreting cytokines such as IFN-γ (28). However, in the keloid environment, this inhibitory effect may be weakened, leading to an abnormal fibrotic response instead.

In addition, the interaction between T cells and other immune cells also has a significant impact on the formation of keloids. For instance, T cells can recruit macrophages and B cells to the damaged area by secreting chemokines, and the activation of these cells can further enhance the inflammatory response (29). Keloid tissue contains a large number of activated macrophages and B cells, which participate in the formation process of keloids through interactions and signal transduction (30).

It is noteworthy that the role of T cells in keloids also involves immune tolerance and autoimmune responses (31). Patients with keloids may exhibit abnormal autoimmune reactions, which could result from T cells incorrectly recognizing or being overly activated by self-antigens. This phenomenon not only leads to a persistent inflammatory state but also further promotes the activation of fibroblasts and collagen production.

#### 3.3.3 Mast cell

Mast cells are immune cells derived from bone marrow, widely distributed in tissues such as the skin, respiratory tract, and gastrointestinal tract. They contain abundant granules that store various bioactive substances, including histamine, proteases (such as trypsin and chymotrypsin), heparin, and various cytokines. When mast cells are stimulated, these substances can be released into the extracellular matrix, participating in various physiological processes such as local inflammatory responses, tissue repair, and immune regulation. In the pathophysiological process of keloid formation, mast cells influence local fibrosis by releasing these inflammatory mediators and cytokines. They are also regarded as important immune cells in the formation of keloids (32).

Mast cells trigger local inflammatory responses by releasing mediators such as histamine (33). The formation of keloids is often accompanied by a chronic inflammatory response, and mast cells are one of the key participants in this reaction. In the early stages of keloid formation, damage to the local tissue leads to the recruitment and activation of mast cells. Once activated, mast cells release a large amount of inflammatory mediators, such as histamine, leukotrienes, and prostaglandins, which promote vasodilation and increase vascular permeability, resulting in localized edema and the accumulation of extracellular matrix (34). These inflammatory responses not only provide a suitable microenvironment for fibroblast proliferation but also further promote keloid formation by regulating the activity of local immune cells.

Mast cells regulate the activity of fibroblasts directly by releasing various growth factors and cytokines (35). In hypertrophic scar tissues, mast cells often exhibit a co-localized distribution with fibroblasts, suggesting an interaction between the two. Studies have found that mast cells can release various growth factors that promote fibrosis, such as TGF-β, PDGF, and fibronectin, etc (36). These factors can stimulate the proliferation and migration of fibroblasts, as well as induce the synthesis of a large amount of extracellular matrix components, especially collagen. Additionally, the cytokines released by mast cells (such as IL-4, IL-13) can also induce the transformation of fibroblasts into myofibroblasts (37). Myofibroblasts are cells with contractile ability that can further tighten and compress local tissues, exacerbating the proliferation of keloids.

Mast cells in keloids not only play a role in promoting the fibrotic process but may also be important in regulating the local immune microenvironment. Studies have shown that the formation of keloids is closely related to the dysregulation of local immune responses, and mast cells, as immune effector cells, can indirectly affect the occurrence and development of keloids by regulating the activity of other immune cells (such as T cells, macrophages, and dendritic cells) (38, 39). For example, the cytokines and chemokines released by mast cells can recruit and activate T cells, thereby modulating the local immune response. In addition, mast cells can interact directly with T cells through the expression of co-stimulatory molecules on their surface, influencing the activation and differentiation of T cells.

The role of mast cells in the formation of keloids is not limited to promoting fibrosis and inflammation. Recent studies have also found that mast cells may have a negative feedback regulatory effect on scar formation by secreting some anti-fibrotic factors or inhibitory molecules (40). For example, heparin released by mast cells has anti-fibrotic properties and can inhibit the proliferation of fibroblasts and collagen synthesis (41). In addition, mast cells may also suppress local inflammatory responses by releasing anti-inflammatory cytokines (such as IL-10), thereby slowing down the proliferation of keloids (42). Therefore, the role of mast cells in the formation of keloids is complex, with both positive effects in promoting fibrosis and potential inhibitory effects through certain pathways.

Mast cells play an important role in immune regulation during the formation of keloids. Mast cells can recognize and bind specific antigens through their surface receptors, triggering a series of immune reactions. These immune responses not only help clear pathogens and harmful substances from the body but also regulate the immune tolerance of the body to its own tissues. However, during the formation of keloids, the immune regulatory function of mast cells may become abnormal, leading to the body’s immune attack on its own tissues, thereby exacerbating the formation and development of keloids.

#### 3.3.4 Regulatory T cells

Tregs are a subset of CD4+ T cells with immune suppressive functions, primarily maintaining immune homeostasis by inhibiting excessive immune responses. The characteristic molecules of Tregs include the transcription factor Foxp3, cell surface molecules CD25, cytotoxic Tlymphocyte-associated antigen 4(CTLA-4), among others. Tregs exert immune suppression through various mechanisms, such as secreting immunosuppressive cytokines (such as IL-10, TGF-β) to inhibit the activity of effector T cells, or directly interacting with antigen-presenting cells to reduce the intensity of immune responses (43).

Tregs play a crucial role in protecting self-immunity in the body, preventing excessive immune responses that could cause tissue damage. However, under certain pathological conditions, the function of Tregs may be erroneously activated or suppressed, leading to inappropriate tissue repair reactions. For example, excessive activation of Tregs may impair the immune system’s monitoring and clearance functions against tumor cells, promoting tumor growth. Similarly, abnormalities in Tregs function may play an important role in the formation of keloids (44).

##### 3.3.4.1 The regulatory role of Tregs on fibroblasts

Tregs regulate the activity of fibroblasts by secreting TGF-β (45). TGF-β is a cytokine with dual roles in the wound healing process. On one hand, it promotes fibroblast proliferation and collagen synthesis in normal healing; on the other hand, overexpression of TGF-β may lead to sustained activation of fibroblasts and abnormal formation of scar tissue (46). The role of Tregs in keloids may involve influencing the behavior of fibroblasts by regulating the TGF-β signaling pathway, thereby promoting or exacerbating the formation of keloids (47).

##### 3.3.4.2 Tregs and the regulation of inflammatory responses

Tregs suppress inflammatory responses by secreting immunosuppressive cytokines such as IL-10 (48). IL-10 is an important anti-inflammatory factor that can inhibit the activity of inflammatory cells such as macrophages, dendritic cells, and T cells, thereby alleviating local inflammation. Tregs directly inhibit the production of TGF-β by releasing IL-10. In patients with keloids, changes in the quantity and function of Tregs may lead to local immune dysregulation, causing ineffective termination of inflammatory responses. This sustained inflammatory response can not only result in abnormal scar tissue formation but also trigger persistent symptoms such as itching and pain.

##### 3.3.4.3 The interaction of Tregs with the local immune microenvironment

The interaction of Tregs with the local immune microenvironment plays a crucial role in the wound healing process, regulating local immune responses (49). Studies have shown that significant changes may occur in the local immune microenvironment in keloids. For example, local macrophages and dendritic cells may be reprogrammed into a pro-fibrotic phenotype, thereby promoting excessive fibroblast activity (50).Tregs regulate the function of macrophages and dendritic cells through their immunosuppressive function (51). Under normal circumstances, Tregs can inhibit the activity of these antigen-presenting cells and reduce the release of pro-fibrotic factors. However, in patients with keloids, the immune regulatory function of Tregs may be impaired, leading to a sustained pro-fibrotic state in the local immune microenvironment. These changes in the local immune microenvironment are one of the key factors contributing to the formation of keloids.

However, although some microenvironmental factors may disrupt the balance of TGF -β/IL-10, the precise regulatory mechanism of Tregs’ dual function and the key critical points that facilitate their pro fibrotic activity in keloids are still not fully understood. The sustained pro fibrotic signaling through chronic activation of the TGF -β/Smad and PI3K/Akt/mTOR pathways may create a self-reinforcing loop that promotes the development of Tregs towards a high TGF - β and low IL-10 phenotype (52). Abnormal interaction with M2 macrophages, fibroblasts, and keratinocytes, mediated by cytokines such as IL-6 and IL-13, may further distort Treg function by inhibiting IL-10 production or enhancing TGF - β secretion (53, 54). In addition, epigenetic or metabolic reprogramming within the keloid niche may lock Tregs in a pro fibrotic state, coupled with potential changes in Treg subpopulation composition that favor TGF -β- mediated inhibition rather than IL-10-dependent regulation (52–54). Interpreting how these factors collectively disrupt Treg homeostasis is crucial for developing therapies that restore immune balance and combat keloid fibrosis. However the precise regulatory mechanisms underlying the paradoxical dual roles of Tregs in fibrosis particularly the critical tipping points that determine their pro-fibrotic versus anti-fibrotic functions remain poorly understood and represent a significant knowledge gap in keloid pathogenesis future research should prioritize single-cell analyses to dissect Treg heterogeneity in keloid niches and investigate how microenvironmental cues such as chronic TGF-β exposure or metabolic reprogramming bias Tregs toward pathogenic phenotypes.

### 3.4 Stem cells

Stem cells are a type of primitive cell with self-renewal ability and multi-directional differentiation potential. According to their differentiation potential and sources, stem cells can be divided into embryonic stem cells (ESCs), adult stem cells, and induced pluripotent stem cells (iPSCs). ESCs have the ability to differentiate into all types of cells in the body. Adult stem cells such as mesenchymal stem cells (MSCs) have multi-directional differentiation abilities, broad sources, and advantages in self-renewal, immune regulation, secretion of growth factors, etc., showing great potential in the field of tissue regeneration and repair (55). iPSCs are reprogrammed from somatic cells *in vitro* and possess pluripotency similar to that of embryonic stem cells, but their clinical application still faces technical bottlenecks and safety issues.

Stem cells, especially MSCs, have been widely studied for the treatment of various fibrotic diseases (56). MSCs not only have immunomodulatory and anti-inflammatory properties but also regulate the local cellular environment, inhibit fibrosis occurrence and development by secreting various growth factors and cytokines through paracrine mechanisms (57). Their application in keloids mainly manifests in the following aspects:

#### 3.4.1 The anti-fibrotic effects of stem cells

The main characteristic of keloids is the abnormal proliferation of fibroblasts and excessive deposition of collagen. In normal skin repair processes, fibroblasts secrete an appropriate amount of collagen to repair tissue. However, in the formation of keloids, this process is abnormally activated, leading to the deposition of excessive collagen proteins (especially type I and type III collagens). Stem cells, especially MSCs, inhibit the excessive activation and proliferation of fibroblasts through various pathways. Studies have shown that MSCs can degrade excessive collagen and inhibit the proliferation of fibroblasts in keloids by secreting anti-fibrotic factors such as tumor necrosis factor-α-induced protein 6 (TSG-6) and matrix metalloproteinases (MMPs) (58). Additionally, stem cells can regulate the TGF-β/Smad signaling pathway to suppress key fibrotic signaling processes in the formation of keloids. TGF-β plays a crucial role in the pathological process of keloids, promoting fibroblast proliferation and collagen synthesis. The presence of MSCs can inhibit this fibrotic process by reducing the activation of the TGF-β signaling pathway (59).

#### 3.4.2 The immunomodulatory role of stem cells

Due to their strong immunomodulatory capabilities, MSCs can regulate local and systemic immune environments through various pathways. Firstly, MSCs can inhibit the pro-inflammatory M_1_ polarization of macrophages towards an anti-inflammatory M_2_ phenotype, thereby reducing the secretion of inflammatory factors and alleviating local inflammation reactions (60, 61). Additionally, MSCs can also secrete various immunomodulatory factors such as IL-10 and prostaglandin E2 (PGE2), which suppress the activation and proliferation of T lymphocytes, regulate Th1/Th2 balance, and restore normal immune system function (62). Therefore, the immunomodulatory effects of stem cells not only alleviate chronic inflammation during the formation of keloids but also inhibit the ongoing development of fibrosis.

#### 3.4.3 The anti-inflammatory effects of stem cells

Inflammatory response is an important factor in the formation of keloids. After skin tissue injury, the healing process initiates acute and chronic inflammatory responses. Acute inflammation helps clear pathogens and necrotic tissue. However, if the inflammatory response is excessive or prolonged, it can lead to fibrosis. Patients with keloids typically have persistent chronic inflammation, characterized by continuous infiltration of inflammatory cells and high expression of pro-inflammatory factors.

Stem cells, especially MSCs, exert significant anti-inflammatory effects through the secretion of anti-inflammatory factors. Studies have shown that MSCs can inhibit the expression of pro-inflammatory cytokines (such as TNF-a, IL-6, IL-1β, etc.) and reduce the infiltration of inflammatory cells by secreting molecules like PGE2, TSG-6, interleukin-1 receptor antagonist (IL-1RA). This helps alleviate local chronic inflammation (63). Additionally, stem cells can regulate the polarization state of macrophages, converting pro-inflammatory M1-type macrophages into anti-inflammatory M_2_-type macrophages, further mitigating the inflammatory response.

#### 3.4.4 The paracrine effects of stem cells and the role of extracellular vesicles

The role of stem cells in keloids is not only dependent on their differentiation potential but also closely related to their powerful paracrine effects. Stem cells regulate local cell behavior and tissue microenvironment by secreting a series of bioactive molecules such as growth factors, cytokines, chemokines, etc (64). Particularly in the treatment of keloids, MSCs can promote normal tissue repair and regeneration, reduce abnormal fibrotic reactions by secreting VEGF, fibroblast growth factor (FGF), insulin-like growth factor (IGF), and other factors. Additionally, the exosomes secreted by stem cells, as a type of extracellular vesicle, have shown potential in the treatment of keloids. Exosomes carry various functional molecules such as proteins, mRNA, miRNA, etc., which can be uptaken by surrounding cells to regulate cell growth, differentiation, and matrix synthesis (65). Through these paracrine mechanisms, stem cells can effectively inhibit the formation of keloids and promote the repair of damaged tissues.

In general, stem cells, especially MSC, exhibit immense potential in the treatment of keloids due to their multilineage differentiation potential, anti-fibrotic, anti-inflammatory, and immunomodulatory effects. With the continuous advancement of cell therapy technologies, stem cells are expected to become an effective and safe approach for treating keloids in the future.

## 4 The signaling pathways related to keloids

### 4.1 TGF-β signaling pathway

TGF-β is a multifunctional cytokine belonging to the TGF-β superfamily. It is widely present in various tissues and cells, with diverse biological functions including regulating cell proliferation, differentiation, migration, apoptosis, and extracellular matrix (ECM) synthesis and degradation. TGF-β has three main subtypes: TGF-β1, TGF-β2, and TGF-β3, with TGF-β1 being the most extensively studied in keloids (66).

In keloids, the immune mechanisms of TGF-βmainly function through the following aspects.

#### 4.1.1 Regulating immune cell function

TGF-β regulates various immune cells, including macrophages, lymphocytes, and dendritic cells. In keloids, TGF-β inhibits the activation of macrophages, reducing the release of inflammatory mediators such as INF-γ, IL-17, thereby alleviating local inflammation. Additionally, TGF-β promotes the generation and activation of Tregs while inhibiting the proliferation of B lymphocytes and antibody production (67). Tregs play a crucial role in immune regulation by suppressing excessive immune responses. However, in keloids, an excessive number of Tregs may lead to immune dysregulation.

#### 4.1.2 Modulating cytokine secretion

TGF-β can influence the secretion of various cytokines, such as interleukins (IL) and tumor necrosis factor (TNF). In keloids, TGF-β upregulates the expression of anti-inflammatory cytokines like IL-10 while downregulating the levels of pro-inflammatory cytokines such as IL-6 and TNF-α, thus regulating the local immune microenvironment.

#### 4.1.3 Regulating immune response reactions

TGF-β plays a crucial role in immune response reactions. On one hand, TGF-β can inhibit the proliferation and activation of Th1 cells while promoting the development and differentiation of Th2 cells, thereby regulating the Th1/Th2 immune balance (68). On the other hand, TGF-β can induce the production and activation of regulatory T cells (Tregs), enhancing their immunosuppressive function to inhibit aberrant immune responses in keloids.

#### 4.1.4 The signaling transduction mechanism of TGF-β in keloids

The Smad signaling pathway is one of the main downstream signaling pathways of TGF-β. In keloids, TGF-β binds to receptors on the cell membrane, activates receptor-regulated Smad proteins such as Smad2 and Smad3, forming a Smad complex that translocates to the cell nucleus to regulate gene expression (69). In addition to the Smad signaling pathway, TGF-β can also activate various non-Smad signaling pathways such as the MAPK signaling pathway, PI3K/Akt signaling pathway, etc., to exert its biological effects in keloids (70, 71). The activation of these signaling pathways can interact and influence each other, collectively participating in the occurrence and development of keloids.

Therefore, TGF-β in the immune mechanism of keloids plays a role in promoting immune cell infiltration, regulating immune cell function, promoting fibroblast activation and collagen deposition, enhancing mast cell function, and other aspects, affecting the formation and development of keloids.

### 4.2 The PI3K/Akt/mTOR signaling pathway

The PI3K/Akt/mTOR signaling pathway plays a crucial role in cell growth, proliferation, metabolism, and apoptosis. This pathway is activated by various upstream signals (including growth factors, extracellular matrix, etc.), promoting the activation of PI3K, and subsequently activating downstream target mTOR through phosphorylation of Akt, regulating protein synthesis, cell metabolism, and proliferation. In keloids, abnormal activation of the PI3K/Akt/mTOR signaling pathway is considered a key factor leading to excessive proliferation of fibroblasts and increased collagen synthesis (72). Several studies using specific inhibitors (such as LY294002 for PI3K and rapamycin for mTOR) or genetic approaches have demonstrated that suppression of this pathway significantly reduces fibroblast proliferation and collagen production both *in vitro* and in animal models (73). These findings highlight the pivotal role of PI3K/Akt/mTOR signaling in keloid pathogenesis and its potential as a therapeutic target (74, 75).

Additionally, the PI3K/Akt signaling pathway also participates in regulating the infiltration and activation of immune cells, especially T cells and macrophages (76). These immune cells play a critical role in the formation and development of keloids. For example, activation of the PI3K/Akt signaling pathway can enhance the function of Tregs, promote the expression of TGF-β1 and collagen derived from Tregs, thereby regulating the activation of fibroblasts and fibrosis. The PI3K/Akt pathway also closely interacts with the TGF-β signaling pathway, synergistically promoting the formation of keloids (77).

However, critical evaluation reveals the limitations of current evidence. Most mechanism studies rely on *in vitro* models (such as single culture of scar tissue fibroblasts) and rodent xenografts, which may not fully summarize the pathophysiology of human scar tissue. There are conflicting data regarding pathway specificity: although rapamycin (mTOR inhibitor) continuously inhibits collagen synthesis, PI3K/Akt inhibitors (such as LY294002) show different therapeutic effects in different scar tissue fibroblast lineages, indicating that cell heterogeneity can affect treatment response (73).

### 4.3 The Wnt/β-catenin protein signaling pathway

The Wnt signaling pathway is a highly conserved signaling pathway that plays an important role in embryonic development, tissue homeostasis maintenance, and the occurrence and development of various diseases. The Wnt signaling pathway mainly includes the canonical Wnt/β-catenin pathway and the non-canonical Wnt pathway. In particular, the canonical Wnt/β-catenin pathway plays a key role in processes such as cell proliferation, differentiation, and migration. Studies have shown that the expression levels of Wnt proteins and their downstream signaling molecules in hypertrophic scar tissues are significantly higher than in normal skin tissues. Specifically, members of the Wnt protein family such as Wnt3a, Wnt5a, etc., are highly expressed in keloids. These proteins activate the β-catenin signaling pathway, thereby regulating processes such as proliferation, differentiation, and migration of fibroblasts in keloids (78).

#### 4.3.1 Regulating the infiltration of immune cells

During the formation of keloids, inflammation occurs, and various immune cells such as macrophages, lymphocytes, etc., are involved. Studies have found that the Wnt signaling pathway plays a regulatory role in the infiltration of immune cells (76, 79, 80). Specifically, Wnt proteins can regulate the expression of chemokines, attracting immune cells to migrate to hypertrophic scar tissues. For example, Wnt3a can upregulate the expression of the chemokine CCL2, promoting the aggregation of macrophages in hypertrophic scar tissues. These immune cells play an important role in the formation of keloids, and the degree of their infiltration is closely related to the severity of keloids.

#### 4.3.2 Influencing the function of immune cells

In addition to regulating the infiltration of immune cells, the Wnt/β-catenin protein signaling pathway can also directly influence the function of immune cells. In hypertrophic scar tissues, Wnt proteins can activate the β-catenin signaling pathway to regulate processes such as proliferation, differentiation, and activation of immune cells (81). For example, Wnt5a can activate the polarization process of macrophages through the non-canonical Wnt pathway, causing them to differentiate into M2-type macrophages. M2-type macrophages play a role in promoting tissue repair and fibrosis during the formation of keloids, and an increase in their number may lead to excessive proliferation of keloids.

#### 4.3.3 Regulating the production of inflammatory mediators

During the formation of keloids, the production and release of inflammatory mediators such as TGF-β, PDGF, etc., have a significant impact on the development of keloids. Studies have found that the Wnt signaling pathway can regulate the production of these inflammatory mediators. Specifically, Wnt proteins can upregulate the expression of inflammatory mediators such as TGF-β, PDGF, etc., by activating the β-catenin signaling pathway (82, 83). These inflammatory mediators can further stimulate the proliferation of fibroblasts and collagen synthesis in keloids, leading to the formation and exacerbation of keloids.

### 4.4 The interaction of the Wnt signaling pathway with other signaling pathways

In the process of hypertrophic scar formation, the Wnt signaling pathway does not exist in isolation but interacts closely with other signaling pathways (84). For example, the Wnt/β-catenin pathway can interact with the TGF-β/Smad pathway to jointly regulate the proliferation of fibroblasts and collagen synthesis in keloids. Additionally, the Wnt signaling pathway can also interact with the Notch signaling pathway, forming a complex signaling network that collectively participates in the occurrence and development of keloids.

In summary, the Wnt/β-catenin signaling pathway plays a crucial role in the formation and development of keloids by regulating immune cell infiltration, influencing immune cell function, and controlling the production of inflammatory mediators. As research progresses, therapeutic strategies targeting the Wnt/β-catenin signaling pathway hold promise in providing new insights and methods for the treatment of keloids. However, further research is needed to elucidate the specific mechanisms underlying the role of the Wnt/β-catenin signaling pathway in keloids, in order to better guide clinical treatments.

### 4.5 Notch signaling pathway in the immune mechanisms of keloids

Notch signaling pathway is a highly conserved intercellular signaling pathway that is widely present in various organisms, especially in mammals where it plays crucial roles. This pathway regulates multiple biological processes such as cell proliferation, differentiation, apoptosis, and immune responses through the interaction between Notch receptors on the cell surface and ligands.

The core components of the Notch signaling pathway include Notch receptors (Notch1-4), ligands (Delta-like 1, 3, 4 and Jagged 1, 2), and intracellular signaling molecules (CSL/RBP-JK, Mastermind-like proteins, etc.). Upon binding of Notch receptors with ligands, the Notch receptor undergoes cleavage, releasing the active Notch intracellular domain (NICD). The NICD then translocates to the cell nucleus, where it binds to CSL/RBP-JK and initiates the transcription of downstream genes (85).

#### 4.5.1 Regulating the polarization of macrophages

The Notch signaling pathway plays an important role in macrophage polarization. Studies have shown that activation of the Notch signaling pathway can promote macrophage polarization towards M1 type (86). The Notch signaling pathway regulates macrophage polarization by inhibiting the expression of signal-regulatory protein α (SIRPα). SIRPα plays a key role in M2-type polarization of macrophages, and its expression is dependent on interaction with CD47 and via SHP-1-mediated intracellular signaling. The Notch signaling pathway inhibits the expression of SIRPα through Hes family co-repressors. In addition, the Notch signaling pathway can also affect the polarization process of macrophages by regulating other molecules such as SOCS3, cylindromatosis, and miR-125a (87). These molecules play an important regulatory role in the macrophage polarization process.

#### 4.5.2 Regulating the proliferation and differentiation of T lymphocytes

T lymphocytes play a pivotal role in the immune mechanisms underlying keloid formation, with their proliferation and differentiation being tightly regulated by the Notch signaling pathway. Activation of Notch signaling promotes T cell expansion and modulates subset differentiation, exerting dual effects on scar pathogenesis. On one hand, Delta-like ligands engage Notch1 receptors to initiate the RBP-Jκ-dependent transcriptional program, which synergizes with the TGF-β/Smad3 pathway to upregulate Foxp3 expression and drive regulatory T cell differentiation (88). Concurrently, Notch-mediated recruitment of DNMT1 methyltransferase establishes repressive epigenetic marks at the IL2 and IFNG loci through CpG island hypermethylation, effectively suppressing Th1 lineage commitment (89, 90).

Conversely, keratinocyte-derived Jagged1 ligands binding to Notch3 receptors on T cells trigger distinct epigenetic remodeling. The RBP-Jκ/p300 complex facilitates histone acetylation at the GATA3 locus, marked by increased H3K27ac deposition, which opens chromatin accessibility and establishes Th2-polarizing gene expression. As the master transcriptional regulator of Th2 differentiation, GATA3 directly transactivates IL-4, IL-5, and IL-13 genes, creating a profibrotic cytokine milieu that may contribute to keloid pathogenesis. The pathway orchestrates these fate decisions through sophisticated integration of transcriptional networks and epigenetic modifications, ultimately influencing scar tissue homeostasis (91).

#### 4.5.3 Influencing the activation and proliferation of mast cells

Mast cells contribute significantly to keloid pathogenesis through the release of pro-inflammatory mediators including histamine and leukotrienes, which promote extracellular matrix deposition and fibrotic progression. Emerging evidence indicates that the Notch signaling pathway modulates mast cell activation and proliferation, thereby influencing keloid formation (92). In keloid tissue, Notch pathway activation appears to enhance mast cell-mediated inflammatory responses, though the precise molecular mechanisms warrant further investigation.

Notch signaling orchestrates macrophage polarization through dual regulatory mechanisms. At the transcriptional level, the NICD-RBP-Jκ complex directly activates M2 marker genes (Arg1, Mrcl) while suppressing NF-κB-mediated expression of M1-associated genes (TNF-α, iNOS (93). Epigenetically, Notch signaling facilitates HDAC1 recruitment to the IRF8 locus, resulting in H3K9 deacetylation and subsequent silencing of this critical M1 polarization regulato (94). Within keloid microenvironments, fibroblast-derived Jagged1 sustains Notch2 receptor activation in macrophages, promoting a profibrotic M2 phenotype that exacerbates scar formation (95).

#### 4.5.4 Regulating the secretion of cytokines

The Notch signaling pathway participates in the pathological process of scar tissue by bidirectionally regulating the cytokine network. Research has shown that activation of this pathway in keloid tissue can significantly promote the secretion of key cytokines such as TGF - β and VEGF, which play a central role in abnormal fibrosis and angiogenesis of scar tissue. Of particular note is the positive feedback amplification mechanism between Notch and TGF - β pathway: on the one hand, the intracellular domain NICD of Notch forms a transcription complex with Smad3 protein, directly enhancing the transcriptional activity of TGFB1 gene (96, 97); On the other hand, TGF - β signaling upregulates the expression level of Notch ligand Jagged1 through a Smad4 dependent pathway. This bidirectional molecular loop continuously amplifies pro fibrotic signals, ultimately leading to the sustained progression and difficult to regress pathological features of scar tissue (93). In conclusion, the Notch signaling pathway plays a significant role in the immune mechanisms of keloids. By regulating the polarization of macrophages, proliferation and differentiation of T lymphocytes, activation and proliferation of mast cells, and secretion of cytokines, the Notch signaling pathway may impact the formation and development of keloids. However, many aspects of the specific mechanisms of the Notch signaling pathway in keloids remain unknown. Future research should further explore the detailed mechanisms of the Notch signaling pathway in keloids, particularly its interactions with other signaling pathways and its differential effects in different cell types. Additionally, investigating therapeutic strategies targeting the Notch signaling pathway could lead to the development of new approaches for treating keloids.

### 4.6 Summary

The formation of keloids is a complex process involving the interaction of multiple signaling pathways. Signaling pathways such as TGF-β, PI3K/Akt/mTOR, Wnt/β-catenin, and Notch play crucial roles in the occurrence and development of keloids. These pathways regulate the proliferation, migration, differentiation, and collagen synthesis of fibroblasts through various mechanisms, including modulation of immune cell infiltration, influence on immune cell functions, and control of the production of inflammatory mediators, thus participating in the formation and progression of keloids. Therefore, targeting the abnormal activation of these signaling pathways and developing corresponding inhibitors or treatment methods may provide new strategies for the prevention and treatment of keloids. Future research should further explore the interactions between these signaling pathways, deepen the understanding of the pathogenesis of keloids, and ultimately provide more effective targeted therapies for clinical treatment.

## 5 Conclusion

Scars are a complex dermatopathological condition, and the pathogenesis involves various cell types and pathways in the immune microenvironment. Through current reviews, the important role of the immune microenvironment in scar formation has gradually been clarified. Immune microenvironment-related cells such as macrophages, T cells, B cells, mast cells, keratinocytes, stem cells, etc., are closely related to scar formation. T cells, especially Th2 cells, promote collagen synthesis in fibroblasts by secreting IL-4 and IL-13 (94). Macrophages polarize into M2 type, secrete fibrosis-promoting factors like TGF-β, drive interactions between myofibroblasts, forming a unique immune microenvironment that further regulates scar fibrosis (98). The formation of scars is closely related to abnormal activation of signaling pathways such as TGF-β, PI3K/Akt/mTOR, Wnt/β-catenin, and Notch. These signaling pathways regulate immune cell infiltration, affect immune cell functions, and secretion of various cytokines and inflammatory mediators, such as pro-inflammatory cytokines (e.g., IL-1,IL-6, TNF-α) and growth factors (e.g., TGF-β, VEGF), recruit more immune cells, stimulate fibroblast proliferation, migration, differentiation, and collagen synthesis, thereby influencing the process of scar formation (99).

We have preliminarily revealed the regulatory role of immune microenvironment- related cells and pathways in the pathogenesis of scars, providing multiple potential therapeutic targets. Inhibitors of signaling pathways like TGF-β, PI3K/Akt/mTOR, Wnt/β -catenin, and Notch, as well as immunotherapies regulating the activity of macrophages and T cells in the immune microenvironment, may offer new perspectives for scar treatment. However, these study still has some methodological limitations: the dependence on *in vitro* models may not fully capture the complex *in vivo* microenvironment; animal models often cannot fully reflect the unique pathological characteristics of human keloids; the heterogeneity of clinical samples in terms of stage, location, and patient factors may confuse the results; technical limitations such as batch analysis mask the contribution of specific cell types.

Future research should focus on clarifying the specific roles of different immune cells in scar development and their regulatory mechanisms to develop more effective intervention strategies.

**Figure 1:**
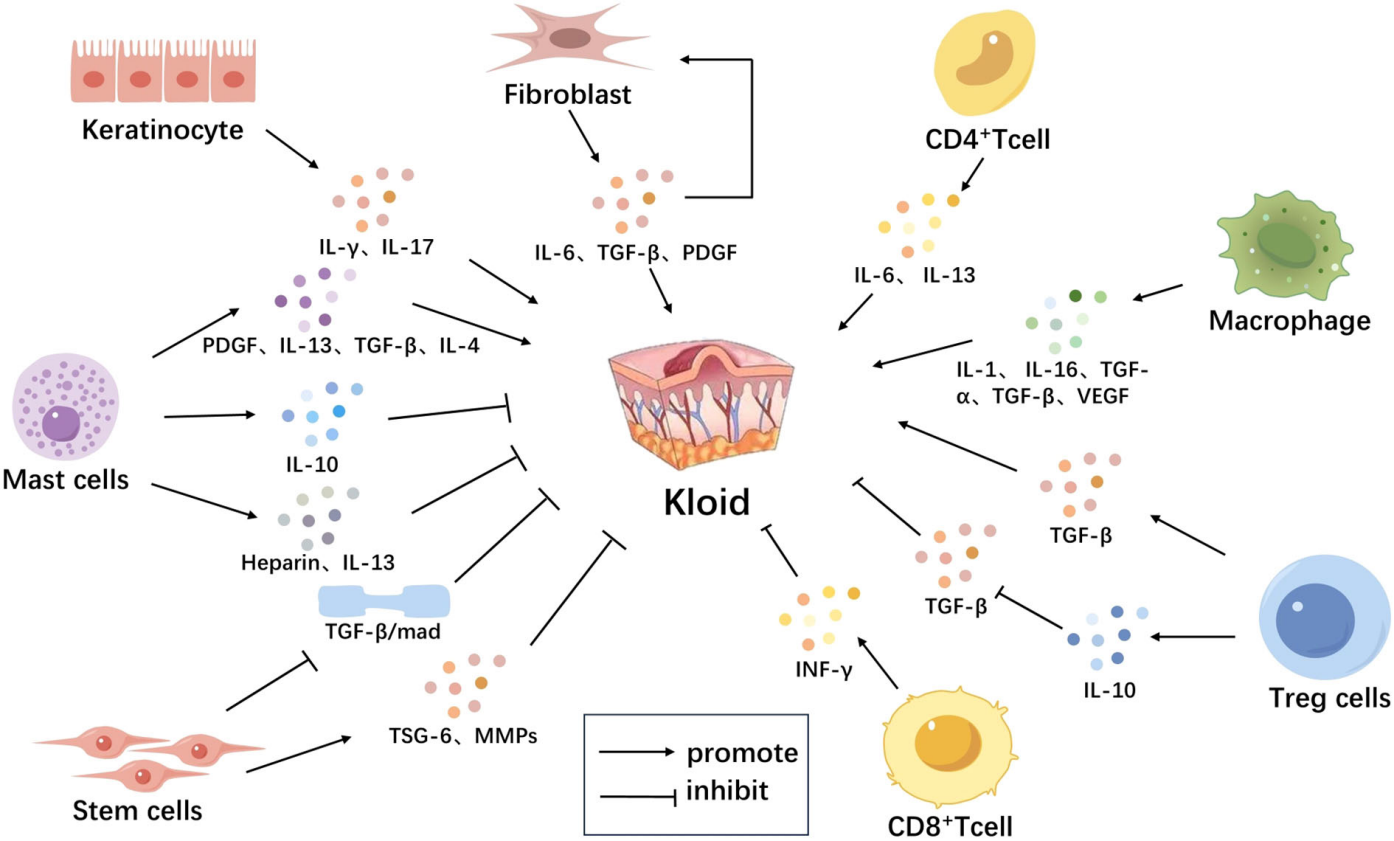
Immune Microenvironment-Related Cells in Scar Tissue. The immune microenvironment-related cells in scar tissue mainly include keratinocytes, fibroblasts, T cells, mast cells, macrophages, and stem cells. Keratinocytes activate T lymphocytes and induce them to secrete various cytokines such as IFN-γ, IL-17, promoting the formation of scars. Fibroblasts secrete IL-6, TGF-β, PDGF, among others, which further stimulate fibroblast proliferation, forming a positive feedback loop leading to continuous growth of scar tissue. Macrophages secrete multiple cytokines such as IL-1, IL-6, TNF-α, TGF-β, VEGF, which recruit more immune cells and stimulate fibroblast proliferation and collagen synthesis, promoting scar formation. CD4+ T cells secrete IL-6, IL-13 to promote scar formation; CD8+ T cells secrete factors like IFN-γ to inhibit scar formation. Mast cells release TGF-β, PDGF, IL-4, IL-13, heparin to promote scar formation; they also release IL-10 to inhibit local inflammatory reactions and slow down scar proliferation. Tregs secrete TGF-β to promote scar formation and secrete IL-10 to inhibit TGF-β secretion and suppress scar formation. Stem cells secrete anti-fibrotic factors such as TSG-6, MMPS, break down excess collagen, and inhibit scar proliferation, also inhibiting scar formation by reducing activation of the TGF-β signaling pathway (**Figure 1**).

**Figure 2:**
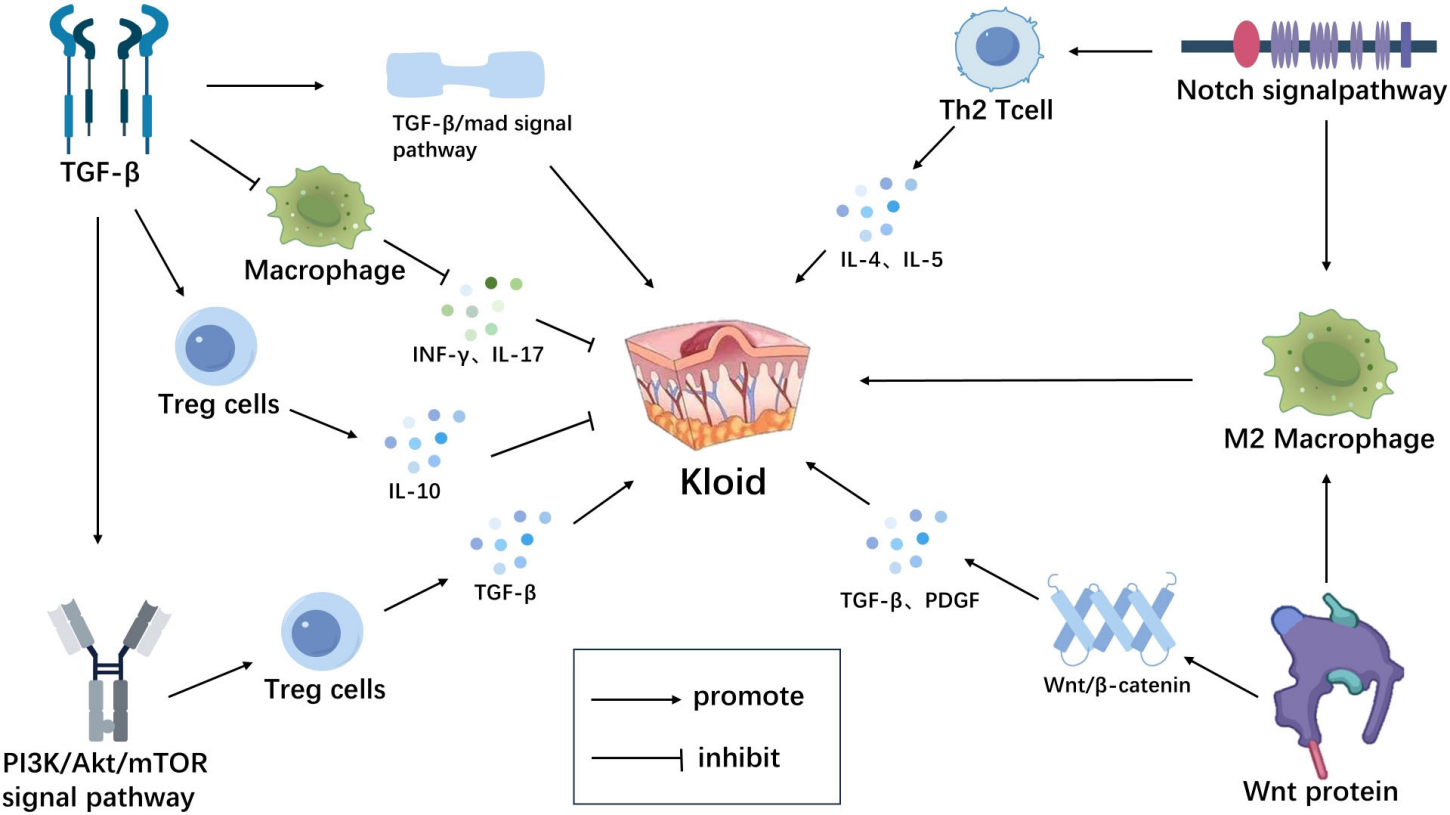
Immune microenvironment-related signaling pathways in scar tissue. TGF-β inhibits macrophage activation, reduces the release of inflammatory mediators (INF-γ, IL-17, etc.) and induces the generation and activation of Tregs, thereby suppressing abnormal immune reactions in scar tissue.By activating the TGF-β/Smad signaling pathway, it promotes scar formation.TGF-β can also activate multiple non-Smad signaling pathways, such as the PI3K/Akt signaling pathway, to exert its biological effects in scar tissue.The PI3K/AKT signaling pathway can promote the expression of TGF-β derived from Tregs, thereby promoting scar formation. Wnt protein activates the β-catenin signaling pathway, upregulates the expression of inflammatory mediators such as TGF-β and PDGF, promoting scar formation. Wnt can also activate the polarization process of macrophages through non-classical Wnt pathways, inducing their differentiation into M2-type macrophages, leading to excessive proliferation of scar tissue. The Notch signaling pathway promotes the differentiation of Th2-type T lymphocytes, secreting cytokines such as IL-4, IL-5, promoting scar formation.It can also promote the polarization of macrophages towards M2 type, leading to scar formation (**Figure 2**). Created with figdraw.com.

## Glossary

IL: interleukin
TGF-β: Transforming growth factor β pathway
PAMPs: pathogen-associated molecular patterns
DAMPs: damage-associated molecular patterns
ILs: interleukins
TNF: tumor necrosis factor
TGF: transforming growth factor
MHC: major histocompatibility complex
ECM: extracellular matrix
MMPs: matrix metalloproteinases
TIMPs: tissue inhibitors of metalloproteinases
PDGF: platelet-derived growth factor
VEGF: vascular endothelial growth factor
DCs: dendritic cells
IFN-γ: interferon-IFN-γ
Tregs: regulatory T cells
CTLA-4: cytotoxic Tlymphocyte-associated antigen 4
ESCs: embryonic stem cells
MSCs: mesenchymal stem cells
IPSCs: induced pluripotent stem cells
TSG-6: Tumor necrosis factor-α-induced protein-6
MMPs: Matrix metalloproteinases
PGE2: Prostaglandin E2
IL-1RA: Interleukin-1 receptor antagonist
FGF: fibroblast growth factor
IGF: insulin-like growth factor.
